# The Linked Dual Representation model of vocal perception and production

**DOI:** 10.3389/fpsyg.2013.00825

**Published:** 2013-11-05

**Authors:** Sean Hutchins, Sylvain Moreno

**Affiliations:** ^1^Rotman Research Institute at Baycrest HospitalToronto, ON, Canada; ^2^Department of Psychology, University of TorontoToronto, ON, Canada

**Keywords:** perception, production, voice, music, pitch, singing, models

## Abstract

The voice is one of the most important media for communication, yet there is a wide range of abilities in both the perception and production of the voice. In this article, we review this range of abilities, focusing on pitch accuracy as a particularly informative case, and look at the factors underlying these abilities. Several classes of models have been posited describing the relationship between vocal perception and production, and we review the evidence for and against each class of model. We look at how the voice is different from other musical instruments and review evidence about both the association and the dissociation between vocal perception and production abilities. Finally, we introduce the Linked Dual Representation (LDR) model, a new approach which can account for the broad patterns in prior findings, including trends in the data which might seem to be countervailing. We discuss how this model interacts with higher-order cognition and examine its predictions about several aspects of vocal perception and production.

## Introduction

One of the most important abilities of humans is the capacity to communicate complex ideas quickly and efficiently. Although there are many ways of communicating with each other, including methods as diverse as body language, signing, and smoke signals, by far the most important medium is the voice. Singing and speech are cultural universals which rely on the voice being physically produced and perceived; these two processes are necessary for communication to occur. Understanding the relationship between vocal perception and production, then, is critical to understanding communication, the nature of the mental processes underlying it, and the most fundamental abilities of humanity.

Singing, even more than speech, has been one of the most profitable places to look for insights into vocal perception and production. On the production side, it involves a similar degree and type of vocal control as speech, and both create a similar type of signal to be perceived by a listener. Furthermore, because of the stylistic communication goals of music, small variations in the produced signal are generally more important than in speech and have thus been the focus of comparatively more research. Since speech and singing both use similar aspects of the vocal signal, the research on perception and production of the voice in a musical context can be informative of how people use their voices in the context of speech. Indeed, many who study this field consider music to have a special relationship with speech processing, due in large part to their overlap and the greater demands of precision of processing in music (see Moreno et al., 2009 or Patel, 2011). This makes singing a particularly interesting and fruitful place to understand the connection (or lack thereof) between perception and production. Furthermore, these findings may shed some insight on how other domains divide processing for these functions.

**Figure 1:**
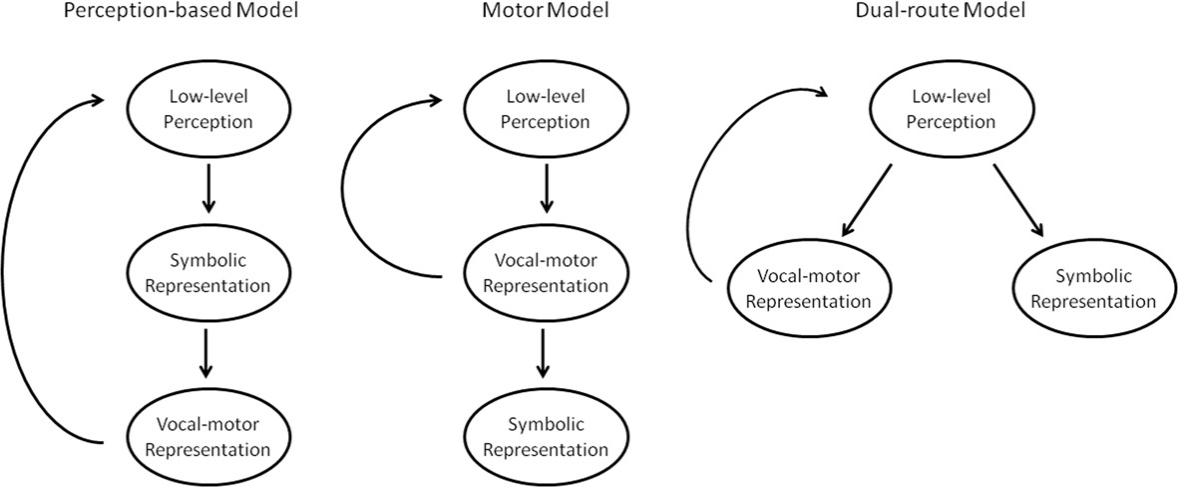
**Three proposed models of perception and production**.

Three basic model architectures have been proposed to explain the relationship between vocal perception and production (Figure 1). The simplest such theory posits that perception necessarily precedes vocal production (Figure 1, left). Thus, when we imitate speech or music, we first construct a symbolic representation of the vocal stimulus. This symbolic representation is then used to construct the vocal-motor representation. These vocal-motor representations are used to issue the appropriate commands to the vocal tract to create the intended sounds. That is, we imitate our symbolic representation of the sound. This model has the benefit of being intuitive and straightforward. It predicts a causal connection between perception and production abilities such that a deficit in our conscious pitch perception abilities would impair our pitch production abilities, while pitch production impairments would not negatively affect our pitch perception abilities.

However, there are alternate models. A motor model of vocal perception (Figure 1, center) would predict the opposite processing stream, where vocal stimuli are first processed for their motor-relevant features, and only afterwards are relayed into our conscious perception for symbolic representation. Such a model preserves the correlation between perception and production, but makes the reverse predictions of the naïve model: vocal production impairments should negatively affect vocal perception abilities, but not vice-versa. Finally, dual-route models (Figure 1, right) predict that vocal stimuli are processed for motor-relevant features and conscious, symbolic representations along two different, independent pathways. This model predicts that vocal perception and production abilities should be uncorrelated, and each can be improved or impaired without affecting the other. These models all have analogues in the speech domain. To take just a few examples, the general auditory account (Diehl et al., 2004), the motor theory of speech processing (Liberman and Mattingly, 1985), and the dual-stream model of speech (Hickok and Poeppel, 2007) mirror the general architectures of the models in Figure 1 from left to right, respectively.

In this review, we will be examining the many factors that affect perception and production abilities, with an eye toward how perception and production might relate to each other and the neural mechanisms underlying each type of ability. We will look at the evidence for each basic type of model and show how different types of evidence point toward structurally different models. Based on this evidence, we introduce the Linked Dual Representation (LDR) model, a synthesis of the relevant features of these prior models that has the potential to explain why vocal perception and production can appear to be both correlated and dissociable abilities. Finally, we will look at the implications and predictions specific to the LDR model and lay out some possible lines of research.

## Production of the singing voice

Anybody who has ever been serenaded by “Happy Birthday” could tell you that there can be quite large individual differences in singing ability. Even among people who have never received any formal music training, we can find both potential future stars and those who cannot seem to find the key. One of the major reasons for individual differences in singing is the fact that singers have such a large number of variables to control simultaneously. To be a good singer, one needs to control the pitch, timbre, timing, and loudness of the voice, with many of these factors changing both between and within individual tones. Of course, part of what makes singing good or bad is culturally-dependent. For example, a Western operatic voice is inappropriate for a Hindustani raga, and vice-versa. Within cultures, too, there are stylistic factors that will affect the judgment of performances- a very skilled country-western singer may sound quite out of place in an R&B recording. Taking stylistic concerns into account, we can identify certain factors that contribute to a good singing performance within particular styles. For example, one of the more well-known and studied of these is the singer's formant. This feature, which is really a compression of the 4th and 5th formants (those regions of the frequency spectrum at which the voice is most resonant; these help define the timbre of the voice) into one large amplitude formant, is a marker of good singing in the Western operatic style (Sundberg, 1987) and is typically achieved by lowering the larynx. Producing a singer's formant can help a solo singer to be heard over an orchestra by concentrating amplitude at frequencies which are not as loud in an orchestra (Sundberg, 1987). Studies of the particular characteristics that make a good vocal style for musical theatre (i.e., belting; Sundberg et al., 1993; Cleveland et al., 2003), country music (i.e., “twang”; Sundberg and Thalén, 2010), and others (Borch and Sundberg, 2011) have also revealed unique techniques for those styles. On the other side of the spectrum, studies of poor singers have found a number of acoustical markers that differentiate them from good singers. These include jitter (which captures irregularity in the microstructure of pitch), shimmer (which captures irregularity in the microstructure of amplitude), and harmonic-to-noise ratio (which captures the strength of harmonic vs. inharmonic frequencies), among others (Titze, 2000; Sataloff, 2005).

However, across all singing styles, one of the most important factors in determining the quality of singing is pitch accuracy. For example, in a study assessing the views of music educators on the singing abilities of non-musicians, intonation (pitch accuracy) was rated as the single most important factor in whether or not a non-musician was perceived as having talent (Watts et al., 2003a). Because of its importance, pitch accuracy is also one of the most widely studied factors in the literature on singing ability (e.g., Dalla Bella et al., 2007; Pfordresher and Brown, 2007; Hutchins and Peretz, 2012a). For example, in a study of untrained singers asked to sing a well-known song in either a city park or a lab setting, Dalla Bella et al. (2007) found a range of singing abilities. These singers showed a great amount of variance in the number of pitch interval errors. All of the participants in the park setting had at least one pitch interval error of greater than a semitone, and a few sang incorrectly on over half of the intervals of the song (there were a total of 31 intervals in the song). Singers performing the same song in a laboratory setting had fewer errors, but nevertheless showed a great deal of variability in performance. Interestingly, the number of errors in the time dimension was much lower across all participants in both groups, indicating that timing accuracy does not seem to be as indicative of singing ability as pitch accuracy.

In another study of note, Pfordresher and Brown (2007) studied singers performing single pitches, single intervals, and short melodies. This study also found a range of abilities on each task, with most being able to sing with an average pitch within one semitone of a target pitch, but some being very inaccurate, as high as 250 cents in error (1 semitone = 100 cents). Their results also indicated that poor pitch singers tend to be inaccurate both in single tones and in intervals and melodies. Poor singers tended to compress intervals. A further investigation (Pfordresher et al., 2010) demonstrated the variability of both single tone and interval tuning, even within individual singers. Here, over 50% of participants showed a standard deviation of greater than 100 cents in their singing, indicating wide-spread imprecision and considerable variability both within and between singers. Numerous other studies have looked at pitch-related singing abilities in the population; these have found consistent variation within non-musicians and consistently better pitch abilities in musicians than non-musicians (e.g., Amir et al., 2003; Watts et al., 2003b; Demorest and Clements, 2007; Nikjeh et al., 2009; Hutchins and Peretz, 2012a). Pitch matching ability also tends to increase in children during their elementary and middle school years (Green, 1990; Yarbrough et al., 1991). Thus, it seems that there is a wide range of abilities in the general population to produce vocal pitches accurately. This wide range of abilities, in combination with the importance of pitch matching in singing, makes it one of the best ways to study vocal-motor control, providing an insight into the accuracy of individuals' vocal-motor representations.

### Factors affecting singing ability

One of the most common assumptions about singing is that poor perception ability drives poor production ability. If people cannot hear pitches accurately, then it stands to reason that they will be inaccurate at imitating those pitches. This is the prediction of the perception-based model (Figure 1, left). Several studies have investigated this hypothesis, and the evidence is mixed. Using a variety of different singing and pitch perception tasks, some studies have found evidence of a correlation between the two abilities (e.g., Amir et al., 2003; Watts et al., 2005; Moore et al., 2007; Estis et al., 2009, 2011). However, many others, using similar designs, have failed to find a significant correlation (e.g., Bradshaw and McHenry, 2005; Dalla Bella et al., 2007; Pfordresher and Brown, 2007; Moore et al., 2008), which argues more for a dual-route model of perception and production (Figure 1, right), making the overall evidence mixed at best.

Two studies addressing this issue are worth pointing out in particular. First, in one of the few studies to use an experimental design, Zarate et al. (2010a) trained participants to better perceive small variations in pitch in the context of micromelodies. However, although they improved at perception, they did not improve in their abilities to produce these same small pitch changes. They concluded that perceptual training does not aid singing ability, thus contradicting the perceptual-based model. Second, in their 2007 study, Pfordresher and Brown found no correlation between pitch perception abilities and their imitation tasks, nor any problems with vocal pitch range in their sample. Thus, they posited that sensori-motor mismappings were the best remaining explanation for poor singing ability in most cases, such that perceived tones were incorrectly mapped onto motor outputs.

In order to sort out the causes of poor singing ability, Hutchins and Peretz (2012a) used a novel methodology involving a new instrument called a slider. This slider produced a synthesized vocal tone that was subject to many of the same limitations as the human voice, including a very fine scale of pitch control. Instead of using their vocal apparatus, though, the participant played the slider by pressing a finger onto a touch-sensitive strip. Thus, it provided a measurement of pitch matching ability independent of the ability to control one's vocal musculature. Pitch-matching ability on the slider was compared to the ability to vocally match a synthesized vocal tone and a prior recording of one's own voice. Participants who could match the pitch with the slider but not with their voice were thus likely to have a vocal-motor control impairment as their primary cause of singing inaccuracies. Those who could match the pitch with the slider and match the recording of their own voice (which had the same timbre as their attempts to match it), but not the synthesized vocal tone, were likely to have a sensori-motor impairment as their primary cause of singing inaccuracies. These singers had a specific difficulty in translating between the timbre of the synthesized voice and the timbre of their own voice. Because their primary deficit was neither in perceiving the relationships among tones, nor in controlling their vocal muscles, but in connecting their perception to an appropriate production, this is considered to be a type of sensori-motor impairment. Finally, those singers who failed at matching pitch both with the slider and the voice are likely to have a perceptual deficit.

The results showed about 20% of singers had a vocal-motor control impairment, 35% had a sensori-motor (timbre) deficit, and only 5% had a perceptual deficit. Participants were universally better at matching pitch with the slider than with their voice, and the results showed a wide range of singing abilities among non-musicians. Singing ability was not aided by multiple attempts, nor was it improved by a visualization of their produced pitch. Although these results show that perception is not a limiting factor in most people's pitch imitation ability, there was nevertheless a modest correlation among non-musicians (*r* = 0.4) between accuracy on the slider and with their voice. These results point to a strong effect of motor and sensori-motor factors on singing ability, with a moderate influence of perceptual ability. This pattern of results suggests aspects of both the perceptual-based model and the dual-route model of vocal perception and production.

Other studies have also shown effects of the target's timbre on pitch-matching ability. Singers are better able to match the pitch of vocal targets with a similar voice than the pitch of instruments (Watts and Hall, 2008) and better able to match the pitch of their own voice than the pitch of other targets (Moore et al., 2008). Poor singers are especially aided by using a human, rather than synthetic, target pitch (Léveque et al., 2012). Educators also report that children tend to be able to match pitch better when modeling a similar voice (reviewed in Goetze et al., 1990).

A number of functional imaging studies have investigated the brain areas that support singing production. These studies have localized the “singing network,” which includes the auditory cortex, insula, supplementary motor area and anterior cingulated, as well as parts of the motor cortex specific to the mouth/lips and larynx. (Perry et al., 1999; Brown et al., 2004; Özdemir et al., 2006; Kleber et al., 2007). This network is involved in motor production, motor planning of sequences, motor initiation, and articulation.

Singing ability is also reflected in neural activation patterns. For example, as might be expected, highly trained singers show more recruitment of laryngeal and mouth areas of the somatosensory cortex than less-trained singers, an effect related to the amount of singing practice (Kleber et al., 2010). They also show more activation in non-cortical regions, such as the basal ganglia, the thalamus, and the cerebellum (Kleber et al., 2010). Other studies using a pitch-shift paradigm, in which the singer's auditory feedback is manipulated while producing the tones, have shown that experienced singers recruit more areas of the singing network than untrained singers (Zarate and Zatorre, 2008). This methodology has shown a particularly strong role of the dorsal premotor cortex in regulating and controlling responses to auditory feedback; this area is thus thought to be highly involved in the interface between perception and production (Zarate and Zatorre, 2008; Zarate et al., 2010b).

## Perception of the sung voice

### General pitch perception abilities

While there has been a good amount of research on singing ability and the factors underlying singing ability, there has been quite a bit less research done of vocal perception. However, we know a great deal about auditory perception in general. In the case of pitch, we can measure just-noticeable differences (or difference limens); in some cases these can be as low as five cents (Zwicker and Fastl, 1999). Individual differences in pitch difference limens, which can be considerable, could contribute to differences in vocal pitch perception abilities. The timbre of tones can also affect pitch perception abilities. Changes in timbre interfere with pitch judgments (Melara and Marks, 1990a,b,c; Krumhansl and Iverson, 1992), and timbre and pitch have been shown not to be perceptually independent (Melara and Marks, 1990a,b,c; Krumhansl and Iverson, 1992; Pitt, 1994; Warrier and Zatorre, 2002). Musicians seem to be less susceptible to timbral interference of pitch processing, however, (Beal, 1985; Pitt and Crowder, 1992; Pitt, 1994).

There is also considerable variability in preferences and judgments of musical intervals. Listeners will show differences between what they consider to be an acceptably-tuned musical interval or note (Rakowski, 1990; Vurma and Ross, 2006; Hutchins et al., 2012), as well as differences in their identification judgments of intervals (Siegel and Siegel, 1977; Halpern and Zatorre, 1979). There are also individual differences related to musical training in preferences in listening to certain types of consonant vs. dissonant intervals (McDermott et al., 2010).

Experience and training can play a large role in pitch perception ability, as evidenced by the differences between musicians and non-musicians (e.g., Pitt, 1994; Moreno and Besson, 2006; Moreno et al., 2009; McDermott et al., 2010; Hutchins et al., 2012). Even among non-musicians, pitch discrimination abilities can be improved with extra training (Zarate et al., 2010a). Tone-language speakers, too, show better pitch perception abilities, presumably due to their greater experience in pitch processing (Pfordresher and Brown, 2009; Bidelman et al., 2013a). Among bilinguals, there is also evidence of causality running in the opposite direction, such that musical ability is predictive of the ability to discriminate and produce non-native speech sounds, both for linguistic tones (Gottfried et al., 2004; Alexander et al., 2005) and for non-tone phonemes (Slevc and Miyake, 2006). Musically trained participants are also better at detecting pitch changes in speech in a foreign language (Marques et al., 2007).

One of the most important neurological correlates of pitch processing ability is the auditory brainstem response (ABR). This response mimics the pitch and some timbral characteristics of a presented tone (Krishnan, 2007; Skoe and Kraus, 2010) and occurs very early in processing, being recorded typically with less than a 10 ms lag following the stimulus. One characteristic of the ABR that is of particular interest is the fact that trained musicians show a higher-fidelity ABR with a shorter lag than non-musicians; this higher fidelity ABR correlates with better ability to make behavioral pitch judgments (Kraus et al., 2009; Bidelman et al., 2011). This benefit is not limited to musicians but generalizes to other groups with high expertise in pitch, such as tonal language speakers (Krishnan et al., 2008; Bidelman et al., 2013b). Other studies have shown that the ABR preserves timbral characteristics more accurately in people with musical backgrounds (Kraus et al., 2009; Bidelman and Krishnan, 2010; Strait et al., 2012). This early benefit in pitch and timbre perception seems to precede cortical representations of pitch and timbre and may be transformed to a more conceptual-level representation of the response as it is transmitted upwards (Bidelman et al., 2013a). This response most likely occurs before any task-relevant effects have time to affect the neural representation. Thus, the fidelity of the brainstem response is a good candidate to affect the accuracy of both pitch perception and production, and may be an indicator of the earliest level of perceptual processing.

### Congenital amusia

One way of learning about the causes and effects of pitch perception, as well as its relationship to production and to the domain of language, is by looking at cases where pitch perception is compromised. Congenital amusia, which is a neurogenetic disorder (Peretz et al., 2007) characterized by impaired music perception ability in the absence of brain damage or hearing or cognitive impairments (Peretz, 2008), provides this kind of test case. This condition is formally diagnosed by the Montreal Battery of Evaluation of Amusia (MBEA; Peretz et al., 2003). The majority of congenital amusics seem to suffer from a selective pitch perception deficit. Amusics are impaired at detecting pitch changes of less than a semitone (Peretz et al., 2002; Hyde and Peretz, 2004) and distinguishing between rising and falling pitches (Foxton et al., 2004; Liu et al., 2010). Amusics also seem to be somewhat impaired in timbre perception (Tillmann et al., 2009; Marin et al., 2012) and memory for pitch (e.g., Gosselin et al., 2009; Tillmann et al., 2009; Williamson et al., 2010). Their condition often leads to amusics not enjoying or seeking out music. Subjectively, they report that music seems like noise; thus it is reasonable to suspect a vicious circle here, where amusics tend to listen to music less often, thus gaining less experience with processing it, making listening even less rewarding than it otherwise might have been.

As would be expected from this type of condition, amusics are impaired in their singing abilities as well. Congenital amusics are judged as poor singers (Ayotte et al., 2002) and make considerably more pitch errors in singing a well-known song than do matched controls (Dalla Bella et al., 2009; Tremblay-Champoux et al., 2010). They are also well-below controls at matching single pitches (Hutchins et al., 2010). However, there are some signs that amusics are not uniformly poor at singing. Certain amusics seem to sing considerably better than would be predicted by their poor perceptual abilities (Dalla Bella et al., 2009; Hutchins et al., 2010; Tremblay-Champoux et al., 2010), and amusics as a whole are aided when directly imitating a model, rather than singing from memory (Tremblay-Champoux et al., 2010). For example, one amusic, ML, is able to sing an array of songs just as well as or better than unimpaired individuals despite her inability to hear errors in songs. These types of findings suggest that conscious perceptual ability may not be a hard limit on amusics' singing abilities. Further evidence for this and its implications will be reviewed later in this paper.

Anatomic and functional MRI studies have shown several differences between congenital amusics and unimpaired individuals. Congenital amusics typically show reduced white matter in the right inferior frontal gyrus, as well as thicker cortices in both that area and the right auditory cortex (Hyde et al., 2007). There is some evidence that there may be differences between amusics and controls in the left analogues of those regions as well (Mandell et al., 2007). In the right hemisphere, these two regions also show reduced functional connectivity (Hyde et al., 2011), and diffusion tensor imaging has shown reduced anatomical connectivity in the right arcuate fasciculus connecting these two regions (Loui et al., 2009). There is some evidence that different regions of the arcuate fasciculus may correlate with pitch perception ability and the discrepancy between perception and production ability (Loui et al., 2009), but this has yet to be corroborated.

Electrophysiological evidence also supports the relationship between pitch perception abilities and frontal-auditory connectivity. Amusics show a normal mismatch negativity (MMN) response (a pre-conscious response to deviations in sound generated in the auditory cortex, Näätänen et al., 2007) to small deviations in pitch which they are unable to consciously detect (Moreau et al., 2009; Peretz et al., 2009). These same deviations, however, generate no P3b response, normally indicative of attentive processing (Moreau et al., 2013). These components, then, seem to be markers of conscious and unconscious pitch perception ability. Taken together, the evidence indicates that frontal regions, auditory regions, and the connection between them regulate normal pitch perception ability, and that there may be anatomically and functionally distinct regions responsible for conscious and unconscious pitch processing. While the regions and processes investigated in these studies are not voice-specific, this type of pitch processing is likely a precursor to voice specific perception and production abilities, which may also be anatomically and functionally distinct.

### Is vocal pitch perception special?

One possible explanation of amusics' better-than-expected singing abilities is that our ability to perceive vocal pitch (and by extension, the processes underlying this ability) may be different from our ability to perceive the pitch of non-vocal tones, such as instruments or synthesized tones. While it is obvious that we can *distinguish* between the voice and other instruments, not many studies have examined the uniqueness of vocal musical perception. One clue that there may be fundamental differences between vocal and non-vocal pitch perception comes from the tuning perception literature. It has been noticed that pitch errors seem to be less noticeable when produced by a voice than by other instruments (Seashore, 1938; Sundberg, 1979). For example, Lindgren and Sundberg (as cited in Sundberg, 1979, 1982) showed that musically experienced listeners would accept as in-tune up to 50–70 cents of tuning errors in a recording of a highly trained singer. Another study looked at recordings of 10 professional singers performing the same song, and found that listeners were highly variable in their assessments of the tuning, with out-of-tune notes being accepted as in-tune and well-tuned notes sometimes being judged as out-of-tune (Sundberg et al., 1996). In contrast, studies of acceptable tuning in synthesized tones show a much smaller range of acceptable tuning, with listeners accepting only 10–15 cents of error (Fyk- in van Besouw et al., 2008). This seems to indicate that listeners use different criteria when judging the pitch of the voice vs. other instruments.

To investigate this effect in a well-controlled manner, Hutchins and Peretz (2012a) directly compared tuning judgments of real and synthesized voices. Musicians and non-musicians listened to pairs of tones and judged them as the same or different. Listeners were less likely to notice the differences in tuning when the tone pairs were real voices than when they were synthesized voices; this pattern held across musicians and non-musicians. Non-musicians needed the two tones to be 50 cents apart to reliably notice the difference between two real vocal tones, compared with only 30 cents for synthesized vocal tones. This pattern held in musicians as well. Hutchins et al. (2012) found very similar results for tuning judgments of a trained voice vs. a violin and extended these findings to a melodic context. This difference in acceptable and noticeable tuning between voices and other timbres was termed the Vocal Generosity Effect and may be evidence of special processing of voices in a musical context as it is consistent across different voices and instruments.

Different types of tuning errors between vocal and non-vocal stimuli are also found in production. Trained singers tend to show more tuning errors than trained instrumentalists. Trained singers have a propensity to begin a note flat (Seashore, 1938), and analyses of recordings of professional singers show deviations of more than 40 cents, both sharp and flat (Prame, 1997). In contrast, studies of violin and wind instruments show average deviations less than 20 cents. This difference in production ability comes despite the fact that people have considerable amounts of experience using their voice. In experts, though, there is a tendency for instrumentalists to practice much more than vocalists (as the voice tends to tire out after a couple of hours of practice). In addition, singers typically use considerably more vibrato than do performers on other instruments, such as the violin (Prame, 1997; Mellody and Wakefield, 2000). Vibrato is sometimes thought to be a way of hiding tuning errors (Yoo et al., 1998), although listeners are nevertheless capable of making quite accurate tuning judgments even for tones with very high-amplitude vibrato (Shonle and Horan, 1980). However, unlike the case of perception, many of these differences between voice and instruments can be explained by the unique motoric requirements of vocal production, which are substantially different from those required by any other instrument.

If the voice *is* processed differently from other instruments, then we should see special neural processes and regions devoted to vocal perception and production. And indeed, there is evidence for just such effects. Belin et al. (2000) showed evidence for subregions of the auditory cortex particularly sensitive to voice perception, called temporal voice areas. These are located bilaterally along the mid superior temporal sulcus, and respond to the voice independent of its linguistic content. Temporal voice areas become less active as the vocal signal is degraded by filtering, indicating a sensitivity to the quality of the input that was reflected in both fMRI and behavioral voice discrimination judgments. Electrophysiological studies also indicate special processing of the voice, with vocal sounds eliciting a fronto-temporal positivity/occipital negativity when compared to environmental sounds or birdsong, peaking around 200 ms post-stimulus (Charest et al., 2009). Another study found a similar frontal positivity of sung tones compared to instrumental sounds, but a bit later, likely due to the more similar acoustic characteristics of these stimuli (Levy et al., 2001), although an MEG study failed to show any differences between similar types of stimuli (Gunji et al., 2003). To the best of our knowledge, no one has yet run an fMRI study comparing activation from perceiving humming to that of perceiving instruments to look for vocal-specific regions involved in music processing. Given the specificity of the motor demands of singing, we would expect to find some such regions; such an experiment would provide an important contribution to the field.

## The relationship between perception and production

To truly understand the nature of perception and production abilities, it is helpful to examine their relationship to each other, specifically the link between conscious vocal perception acuity and vocal production accuracy. The evidence reviewed so far shows a moderate, but not overwhelming correlation between perception and production abilities, which suggests a connection, rather than dissociation, between the two. This points more toward a perceptual-based or motor model of perception and production, rather than a dual route model (see Figure 1). However, other lines of evidence tend to argue against the simple and motor models, and dual-route models have been suggested to explain this pattern of findings (Griffiths, 2008).

### Perception-production dissociations in congenital amusia

Some of the best evidence arguing for a dual-route model of perception and production comes from congenital amusics. Although most congenital amusics, who have severely impaired pitch perception abilities, are impaired in their singing ability, there is evidence that some amusics nevertheless retain the ability to sing accurately. Dalla Bella et al. (2009) identified three amusics (out of eleven tested) who were unimpaired at singing the correct intervals in a well-known song, including one who was unimpaired even without the aid of the lyrics—a condition in which most amusics fail to complete more than a few notes of the song. Hutchins et al. (2010) tested congenital amusics in a single-pitch matching task and found that despite amusics' overall inaccurate performances, they showed a consistent, linear relationship between the imitations and the target tones.

These studies hint that amusics may demonstrate better overall singing ability than would be predicted from their abilities on perceptual tasks. Recently, a number of studies have attempted to directly compare perception and production abilities in amusia, to serve as direct tests of vocal perception and production models. Loui et al. (2008) presented three amusics with two note sequences and asked amusics to imitate the interval, then to describe whether the second note had been higher or lower than the first. The amusics were impaired at describing the direction of the second note, but they performed similarly to controls at singing an interval that went in the correct direction, although they were still inaccurate at producing an interval of the correct distance.

Some of our recent work also demonstrates a similar discrepancy between pitch perception and production ability in amusics. In one ongoing study (Hutchins and Peretz, 2010), we tested amusics' pitch matching abilities with the slider and a vocal imitation condition (the same as used in Hutchins and Peretz, 2012a, Experiment 1; see above). As expected, amusics as a group performed worse than matched controls at both slider and vocal pitch matching. However, we found two participants who performed at levels comparable to normal participants on the vocal imitation task and, notably, better than their performance on the slider. This is a pattern of results not found among normal participants, who almost invariably show excellent pitch matching performance on the slider, even among non-musicians. This demonstrates that for these two amusics, their vocal pitch matching ability was not constrained by their pitch perception ability, arguing against the perceptual-based model of pitch perception and production.

Another of our studies looked at the pitch shift effect. This effect is an automatic compensatory response to a sudden shift in pitch of the feedback of a sung or spoken utterance. When most participants hear such a shift in their own voice, there is a quick reaction to change the pitch of their voice in the opposite direction. We tested amusics and controls in a pitch shift paradigm, where a pitch shift would occur in the middle of an imitative response. Our results showed that a subset of amusics showed a preserved pitch shift effect, showing normal pitch shift responses to both large (2 semitone) and small (25 cent) shifts. This is strong evidence that amusics do process even small pitch shifts when they are relevant to vocal-motor control. In addition, this study also found evidence of a correlation between the pitch shift effect and pitch matching accuracy (absent of any shift), strengthening the idea that this retained pitch shift response is related to generally preserved vocal-motor control. Together, this presents a strong contrast with amusics' previously documented disabilities in consciously perceiving small pitch changes.

We also see evidence for dissociation of vocal perception and production abilities in amusics' use of pitch in speech. Unlike in tone languages, pitch is non-lexical in most European languages. However, it plays a strong role in prosody and can determine the meaning of certain types of statement/question pairs. Liu et al. (2010) showed that amusics were somewhat poorer than controls at discriminating between statements and questions differing only in pitch contour. However, just as with intervals (Loui et al., 2008), they were better at imitating the pitch contour of these same sentences (although still below the level of matched controls). Hutchins and Peretz (2012b) tested amusics with speech examples containing pitch changes that did not systematically alter the meaning of the sentence. In this experiment, amusics showed an impaired ability to perceive pitch changes between sentences, but no impairment at imitating those same pitch differences, compared to controls. Similarly, in the pitch shift study (Hutchins and Peretz, 2013), we found no difference between pitch shift responses to spoken vs. sung utterances. The fact that pitch perception-production dissociation occurs across music and speech indicates that it is a function of vocal pitch perception and control, rather than a function of music.

Neural evidence also supports the dissociation between pitch perception and production in amusics. Loui et al. (2009) found that pitch perception abilities were correlated with tract density along the superior route of the arcuate fasciculus, whereas the lower route was correlated with the difference between their perception and production abilities. While a somewhat complicated story (all the more so because the association runs in the reverse direction to some other theories of dual-route processing, e.g., Goodale and Milner, 1992; Hickok and Poeppel, 2004), this is the first evidence of direct correlations between these dissociations in amusics and specific neuroanatomical structures.

### Evidence for perception-production dissociations in normal subjects

A few studies have shown similar evidence for dissociations between perception and production abilities in an unimpaired population. In one study, Hafke (2008) used a vocal pitch shift paradigm to test trained singers. She found that they showed a normal pitch shift effect, even when the shifts were so small that the participants were unaware that they had occurred at all. This is similar to the pattern of results found among congenital amusics (Hutchins and Peretz, 2013). Vurma (2010) showed a related effect, demonstrating that trained singers' musical interval production abilities are more finely honed than their abilities to perceive the same intervals. Results such as these indicate that the independence of vocal-motor pitch control from conscious pitch perception is not limited to cases such as amusia, which again argues against a perceptual-based model.

The reverse pattern, better conscious perception than production ability, is even more common in normal participants. Hutchins and Peretz (2012a) showed that almost every participant was more capable of matching pitch with an instrument than with their voice in many cases over an order of magnitude better. This pattern held true for musicians and non-musicians alike and demonstrated that poor vocal pitch accuracy does not lead to poor pitch perception ability, as would be predicted by a motor theory. However, there was a moderate correlation between instrumental and vocal pitch matching abilities, arguing against a dual-route theory. A few other studies have found evidence of such perception-production connections (e.g., Amir et al., 2003; Watts et al., 2005; Moore et al., 2007; Estis et al., 2009, 2011), though others have failed to do so (Bradshaw and McHenry, 2005; Dalla Bella et al., 2007; Pfordresher and Brown, 2007; Moore et al., 2008). The preponderance of evidence shows a weak connection between pitch perception and singing ability, but also indicates that poor pitch perception ability is not necessarily the main cause of poor singing ability.

Similar evidence of this dissociation comes from second language learners. Many late second language learners will gain the ability to comprehend a second language, but will nevertheless be unable to speak it with any degree of fluency. Other second language learners, however, will show an opposite pattern, where their production ability will outstrip their comprehension ability. This latter pattern is typically shown by people who need to perform or deliver information in a second language, such as the singer who performs a Mozart opera without speaking a word of German, whereas the former is more characteristic of an immigrant immersed in a second language who does not have the opportunity or inclination to speak it often. Again, like with pitch in singing, perception and production ability in a second language will broadly correlate, but are nevertheless dissociable abilities.

## The linked dual representation model

**Figure 2:**
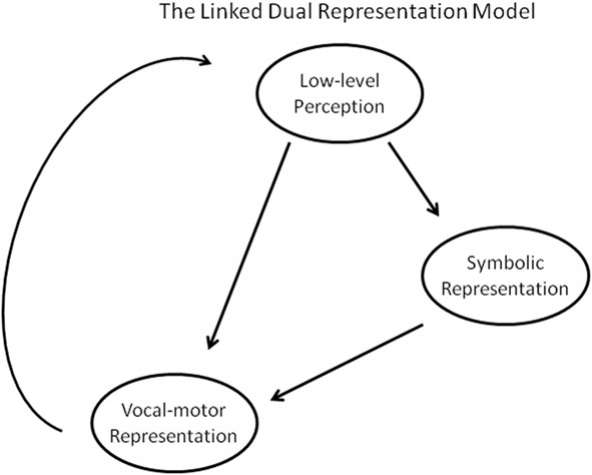
**The Linked Dual Representation model**.

Across these studies, we see two main patterns emerging. First, there is a trend for people who are poor at pitch perception to be worse singers, holding across amusics and unimpaired people. This correlation is not perfect, however, and perception does not determine pitch matching abilities. Second, in many cases, people's production abilities can outstrip their perceptual limitations (or vice versa); this pattern can arise in both perceptually impaired and unimpaired people. To account for these two main patterns we propose a new model of adult human vocal perception and production: The LDR model (Figure 2). Like a dual-route model, the LDR model predicts that vocal information can be processed in two distinct ways. First, it can be encoded as a symbolic representation, such that we gain conscious knowledge of the identifiable features of the vocal stimulus. This process, which is what we normally equate with conscious perception, allows us to determine whether a tone is higher or lower than another, the same or different from another, and allows us to make identification and categorization judgments. Second, vocal information can be encoded as a motoric representation, such that it enables reproduction, imitation, or generative production. The LDR model predicts that vocal information can be directly encoded as a motoric representation, without mediation through a symbolic representation. Just as a point in space can be represented with Cartesian or polar coordinates, each of which is better suited to particular calculations, these symbolic and motor representations support different kinds of behaviors.

However, unlike other dual-route models, the LDR model also predicts that the vocal-motor representation can be mediated by the symbolic representation (see Figure 2). Whereas most dual-route model fail to predict the broad correlation seen between vocal perception and production abilities (e.g., Goodale and Milner, 1992; Griffiths, 2008; Hutchins et al., 2010), this aspect of the model is designed to incorporate this effect. The LDR model predicts that a vocal-motor representation is influenced directly by the low-level perceptual information, but also indirectly by our conscious perception, identification, and category judgments of the information. This is a unidirectional link between the symbolic and vocal-motor representations; the latter cannot directly affect the former. Finally, there is a process of feedback from production back to low-level perception; this process is taken to reflect both auditory feedback from actual productions as well as efferent feedback from actualized motor plans.

All of these processes are variable in strength and are influenced by top-down mechanisms, similar to the way in which executive function can moderate transfer effects between speech and music (Moreno and Bidelman, 2013). The relative influence of the symbolic and direct motoric encoding of a tone on its production can be mediated by the task requirements and context. Even the degree to which a tone is initially encoded symbolically or motorically is influenced by the intention of the listener. A listener who is tasked with comparing a note to a template or identifying an interval will preferentially encode it symbolically, whereas the same input would lead to a stronger vocal-motor encoding in the context of an imitation task. These effects can be visualized as a change in the relative sizes of the arrows.

This model, although motivated by pitch, is intended to apply to other aspects of vocal processing, including timbre, loudness, and phonemic processing. There is nothing about symbolic representation or motoric encoding which does not apply equally to other aspects of vocal tones. This generalization is motivated by several factors, including amusics' impairment in speech perception but not production (Hutchins and Peretz, 2012b), and variability in speech perception and production abilities among normal participants in contexts such as second language learning. However, the applicability of this model to speech warrants further study. The model assumes that initial perception of these attributes can vary across individuals; this variance is passed along to subsequent steps and can influence the accuracy of both types of encoding. It also assumes that individuals can vary in skill in transforming between these different representations accurately, independently of their initial perceptual abilities. Together, these variances in different abilities can explain the patterns of individual difference in perception, discrimination, and imitation abilities.

Taken together, this model provides a more complete explanation of the data than previously proposed models by combining some of the features of previous models. For example, similar to other dual-route models that have been proposed, the LDR model is able to predict dissociations between perception and production among congenital amusics. This model posits that congenital amusics are impaired at encoding pitch symbolically and are thus poor at tasks such as categorization or identification of pitch. Because symbolic representations are responsible for our awareness of pitch, congenital amusics also have diminished awareness of pitch, leading to their lower enjoyment of music. However, they retain their ability to encode pitch as a vocal-motor code. Thus, in some cases, they retain their ability to imitate pitches and respond to pitch changes, often just as well as normal participants. However, they are still, on average, below the abilities of normal participants, which is due to the lack of contribution from a symbolic representation of pitch. A similar argument using naturally occurring variances in abilities can also explain why normal individuals will occasionally show a similar dissociation between conscious perception and production abilities.

However, straightforward dual-route models are unable to explain cases where there seems to be a relationship between perception and production. In contrast, the influence of the symbolic representation on the vocal-motor encoding in the LDR model allows it to explain the moderate correlation between pitch perception ability and imitation ability. Furthermore, this route of influence also allows us to explain the broad correspondence between what we produce and what we hear- most people's imitative responses broadly line up with their perceptual judgments (although not a one to one correspondence). This processing flow, and the independent variance in these abilities, can explain why individual differences in perception and production abilities co-vary but are not perfectly predictive.

## Future directions

The LDR model makes several predictions, which would be profitable to explore in future research. First, because this model is assumed to apply to all vocal abilities, rather than specifically to the domain of music or speech, this model predicts that vocal perception and production abilities should be domain-independent. We would expect to find that, in general, people who are better at singing should be better at using their voice for speaking and vice-versa. It has already been shown that congenital amusics are unimpaired at speech imitation (Hutchins and Peretz, 2012b), and they typically report no general speech production problems. The LDR model predicts that this general phenomenon should carry over to an unimpaired population as well. For example, trained singers should be better at speech imitation, and people skilled at manipulating their voices (such as voice actors) should be better than average at singing. This leads to the interesting prediction that training in singing should also help public speaking ability (above and beyond the benefit of simply becoming more comfortable performing in front of others). Similar relationships should also be found between experts in speech and music perception (such as speech therapists or piano tuners). However, the model also predicts that these abilities are task-dependent—better singers are not necessarily better at perceiving speech sounds. Showing such a pattern would help confirm the domain-generality of this model.

A particularly interesting aspect of this prediction arises when considering the case of dyslexia, which is fundamentally an impairment in reading and writing skills. Many instances of dyslexia are assumed to arise from an impairment of phonetic abilities (Bradley and Bryant, 1978, 1983; Bruck, 1992), which can be considered to be difficulty forming an adequate motor representation of speech sounds (Heilman et al., 1996; Hickok and Poeppel, 2004; D'Ausilio et al., 2009). The LDR model bears a few similarities to dual-route models of sentence reading, which assume that phonological and whole-word routes are mediated by separate neural pathways (e.g., Coltheart et al.'s Dual Route Cascade model, 1993). Both models explain dyslexics' particular difficulties with reading non-words. However, the LDR model puts the phonological difficulties of dyslexics in the context of a general impairment of vocal-motor encoding. Because of this, we would predict that dyslexics should be worse than non-dyslexics at tasks requiring speech imitation and that they would be particularly influenced by the mediating influence of the symbolic representation of phonemic sounds. Thus, dyslexics should be particularly sensitive to the categorical representations of sounds and less able than non-dyslexics at imitating within-category variations in speech sounds.

Another unique prediction of the LDR model comes from taking the dynamics of the system into account. Although a production response can be constructed directly from the input or mediated by the symbolic encoding of the input, the latter route to motor responses involves more steps and would thus take more time to perform. This explains several interesting facts about the timing of vocal responses. In the pitch shift task, for example, responses occur very rapidly and automatically, typically around 100–200 ms after the pitch shift. However, when asked to consciously control the pitch shift response (by inhibiting it, for example), participants are unable to do so as quickly and take another 200–300 ms to make a conscious adjustment to their automatic shift response (Burnett et al., 1998). Our model posits that the controlled response must come through conscious awareness via a symbolic representation of vocal pitch, whereas the automatic response comes directly from a motor-representation of the feedback, creating the different time courses of the two responses.

A similar effect can be found in speech shadowing. Listeners have the ability to shadow a stream of speech (e.g., Chistovich, 1960; Chistovich et al., 1960; Marslen-Wilson, 1973) with a delay as short as 150 ms. While both close and distant shadowing can be quite accurate, and are subject to the same global effects of context (Marslen-Wilson, 1973, 1985), those who shadow speech quickly typically report that they were repeating the material “before they understood [it]” (Chistovich et al., 1960, see also Marslen-Wilson, 1985), whereas the distant shadowers reported knowing what the words were before repeating them. Marslen-Wilson (1985) described evidence that, in certain cases, distant shadowers were more affected by the meaning of words than close shadowers, a fact that makes sense if close shadowers were using a direct encoding from vocal input to vocal motor code and distant shadowers made use of the slower route through symbolic representation of words in their shadowing. Interestingly, when close shadowers were forced to consider the meaning of the words they were shadowing, their performance became slower, more like that of close shadowers (Marslen-Wilson, 1985), a process which can also be explained by the latency of the two analysis paths. Our model would also make the counterintuitive prediction that variation in the speech sounds, such as in different regional accents, would be more likely to be preserved in close shadowers than distant shadowers, due to the normalization process inherent in creating symbolic representations of the stream of speech.

These dynamical properties of the model could be tested directly using absolute pitch possessors. We would predict that in a vocal matching task, requiring a speeded response would make more use of the direct route to a vocal-motor encoding, bypassing the symbolic representation of pitch. However, forcing a delayed response (past the length of the sensory buffer) would lead to greater mediation of the symbolic representation. Because absolute pitch listeners are able to categorize pitches into distinct pitch classes (Takeuchi and Hulse, 1993; Levitin and Rogers, 2005), we would expect that these listeners would be more influenced by their categorizations when making delayed responses, whereas non-absolute pitch listeners should merely show a general decrease in accuracy over longer timescales (as in Estis et al., 2009).

One final avenue worth considering is the connection between the LDR model and the mirror neuron system. This system, which is hypothesized to underlie our abilities to recognize the connections between our actions and those of others (Rizzolatti et al., 2001; Kohler et al., 2002; Rizzolatti and Craighero, 2004), may be of great importance in the ability to imitate others' actions (Brass and Heyes, 2005; Heyes, 2011) and may play a role in speech processing as well (Rizzolatti and Arbib, 1998; although the importance of mirror neurons is not universally agreed upon, see Hickok, 2009, for example). The LDR model's ability to represent an input as a motor code and a symbolic code may be related to the mirror neuron system's purported ability to mediate between these two codes, and it may well be that dissociations between perceptual and production abilities are more likely to be found in people with poorer mirror neuron systems. As both of these models intend to describe the relationship between perception and imitation tasks, further research into their connection (or lack thereof) could be very revealing.

## Conclusion

There is a great deal of variability in vocal perception and performance abilities and only a modest correlation between the two. Vocal perception and production are highly related to speech and musical processing, and we see evidence of a relationship in abilities between the two domains. However, despite the link between vocal perception and production abilities, there is growing evidence supporting a dissociation between them, both in impaired and unimpaired individuals. The LDR model can explain both these broad trends in the data and makes several new predictions about speech imitation, singing, and response timing. We believe this model will help to interpret a wide variety of experiments and can create a common framework for understanding vocal perception and production.

### Conflict of interest statement

The authors declare that the research was conducted in the absence of any commercial or financial relationships that could be construed as a potential conflict of interest.

## References

[B1] Alexander J. Wong P. C. M. Bradlow A. (2005). Lexical tone perception in musicians and nonmusicians, in Proceedings of Interspeech 2005 - Eurospeech - 9th European Conference on Speech Communication and Technology (Lisbon), 397–400

[B2] Amir O. Amir N. Kishon-Rabin L. (2003). The effect of superior auditory skills on vocal accuracy. J. Acoust. Soc. Am. 113, 1102–1108 10.1121/1.1536632 12597203

[B3] Ayotte J. Peretz I. Hyde K. (2002). Congenital amusia: a group study of adults afflicted with a music-specific disorder. Brain 125, 238–251 10.1093/brain/awf028 11844725

[B4] Beal A. L. (1985). The skill of recognizing musical structures. Mem. Cogn. 13, 405–412 10.3758/BF03198453 4088050

[B5] Belin P. Zatorre R. J. Lafaille P. Ahad P. Pike B. (2000). Voice-selective areas in human auditory cortex. Nature 403, 309–312 10.1038/35002078 10659849

[B6] Bidelman G. M. Hutka S. Moreno S. (2013a). Tone language speakers and musicians share enhanced perceptual and cognitive abilities for musical pitch: evidence for bidirectionality between the domains of language and music. PLoS ONE 8:e60676 10.1371/journal.pone.0060676 23565267 PMC3614545

[B9] Bidelman G. M. Moreno S. Alain C. (2013b). Tracing the emergence of categorical speech perception in the human auditory system. Neuroimage 79, 201–212 10.1016/j.neuroimage.2013.04.093 23648960

[B7] Bidelman G. M. Gandour J. T. Krishnan A. (2011). Cross-domain effects of music and language experience on the representation of pitch in the human auditory brainstem. J. Cogn. Neurosci. 23, 425–434 10.1162/jocn.2009.21362 19925180

[B8] Bidelman G. M. Krishnan A. (2010). Effects of reverberation on brainstem representation of speech in musicians and non-musicians. Brain Res. 1355, 112–125 10.1016/j.brainres.2010.07.100 20691672 PMC2939203

[B10] Borch D. Z. Sundberg J. (2011). Some phonatory and resonatory characteristics of the rock, pop, soul, and swedish dance band styles of singing. J. Voice 25, 532–537 10.1016/j.jvoice.2010.07.014 20926250

[B11] Bradley L. Bryant P. (1978). Difficulties in auditory organization as a possible cause of reading backwardness. Nature 271, 746–747 10.1038/271746a0 625341

[B12] Bradley L. Bryant P. (1983). Categorizing sounds and learning to read—A causal connection. Nature 310, 419–421 10.1038/301419a0

[B13] Bradshaw E. McHenry M. A. (2005). Pitch discrimination and pitch matching abilities of adults who sing inaccurately. J. Voice 19, 431–439 10.1016/j.jvoice.2004.07.010 16102669

[B14] Brass M. Heyes C. (2005). Imitation: is cognitive neuroscience solving the correspondence problem. Trends Cogn. Sci. 9, 489–495 10.1016/j.tics.2005.08.007 16126449

[B15] Brown S. Martinez M. J. Hodges D. A. Fox P. T. Parsons L. M. (2004). The song system of the human brain. Cogn. Brain Res. 20, 363–375 10.1016/j.cogbrainres.2004.03.016 15268914

[B16] Bruck M. (1992). Persistence of dyslexics' phonological awareness deficits. Dev. Psychol. 28, 874–886 10.1037/0012-1649.28.5.874

[B17] Burnett T. A. Freedland M. B. Larson C. R. Hain T. C. (1998). Voice F0 responses to manipulations in pitch feedback. J. Acoust. Soc. Am. 103, 3153–3161 10.1121/1.423073 9637026

[B18] Charest I. Pernet C. R. Rousselet G. A. Quiñones I. Latinus M. Fillion-Bilodeau S. (2009). Electrophysiological evidence for an early processing of human voices. BMC Neurosci. 10:127 10.1186/1471-2202-10-127 19843323 PMC2770575

[B19] Chistovich L. A. (1960). Classification of rapidly repeated speech sounds. Akusticheskii Zh. 6, 392–398

[B20] Chistovich L. A. Aliakrinskii V. V. Abulian V. A. (1960). Time delays in speech repetition. Vopr. Psikhol. 1, 114–119

[B21] Cleveland T. F. Sundberg P. J. Prokop J. (2003). Aerodynamic and acoustical measures of speech, operatic, and Broadway vocal styles in a professional female singer. J. Voice 17, 283–297 10.1067/S0892-1997(03)00074-2 14513952

[B22] Coltheart M. Curtis B. Atkins P. Haller M. (1993). Models of reading aloud: dual-route and parallel-distributed-processing approaches. Psychol. Rev. 100, 589 10.1037/0033-295X.100.4.589

[B23] Dalla Bella S. Giguère J. F. Peretz I. (2007). Singing proficiency in the general population. J. Acoust. Soc. Am. 121, 1182–1189 10.1121/1.2427111 17348539

[B24] Dalla Bella S. Giguère J. F. Peretz I. (2009). Singing in congenital amusia: an acoustical approach. J. Acoust. Soc. Am. 126, 414–424 10.1121/1.3132504 19603898

[B25] D'Ausilio A. Pulvermüller F. Salmas P. Bufalari I. Begliomini C. Fadiga L. (2009). The motor somatotopy of speech perception. Curr. Biol. 19, 381–385 10.1016/j.cub.2009.01.017 19217297

[B26] Demorest S. M. Clements A. (2007). Factors influencing the pitch-matching of junior high boys. J. Res. Music Educ. 55, 190–203 10.1177/002242940705500302

[B27] Diehl R. L. Lotto A. J. Holt L. L. (2004). Speech Perception. Annu. Rev. Psychol. 55, 149–179 10.1146/annurev.psych.55.090902.142028 14744213

[B29] Estis J. M. Coblentz J. K. Moore R. E. (2009). Effects of increasing time delays on pitch-matching accuracy in trained singers and untrained individuals. J. Voice 23, 439–445 10.1016/j.jvoice.2007.10.001 18314306

[B30] Estis J. M. Dean-Claytor A. Moore R. E. Rowell T. L. (2011). Pitch-matching accuracy in trained singers and untrained individuals: the impact of musical interference and noise. J. Voice 25, 173–180 10.1016/j.jvoice.2009.10.010 20456914

[B32] Foxton J. M. Dean J. L. Gee R. Peretz I. Griffiths T. (2004). Characterization of deficits in pitch perception underlying tone-deafness. Brain 127, 801–810 10.1093/brain/awh105 14985262

[B33] Fyk J. (1982). Perception of mistuned intervals in melodic context. Psychol. Music Spec. Ed. 36–41 [cited in Van Besouw et al., 2008]. Available online at: http://psycnet.apa.org/psycinfo/1984-14060-001

[B34] Goetze M. Cooper N. Brown C. J. (1990). Recent research on singing in the general music classroom. Bull. Counc. Res. Music Educ. Counc. 104, 16–37

[B35] Goodale M. A. Milner A. D. (1992). Separate visual pathways for perception and action. Trends Neurosci. 15, 20–25 10.1016/0166-2236(92)90344-8 1374953

[B36] Gosselin N. Jolicoeur P. Peretz I. (2009). Impaired memory for pitch in congenital amusia. Ann. N.Y. Acad. Sci. 1169, 270–272 10.1111/j.1749-6632.2009.04762.x 19673791

[B37] Gottfried T. L. Staby A. M. Ziemer C. J. (2004). Musical experience and Mandarin tone discrimination and imitation. J. Acoust. Soc. Am. 115, 2545 10.1121/1.4783674

[B38] Green G. A. (1990). The effect of vocal modeling on pitch-matching accuracy of elementary schoolchildren. J. Res. Music Educ. 38, 225–231 10.2307/3345186

[B39] Griffiths T. D. (2008). Sensory systems: auditory action streams? Curr. Biol. 18, R387–R388 10.1016/j.cub.2008.03.007 18460320

[B40] Gunji A. Koyama S. Ishii R. Levy D. Okamoto H. Kakigi R. (2003). Magnetoencephalographic study of the cortical activity elicited by human voice. Neurosci. Lett. 348, 13–16 10.1016/S0304-3940(03)00640-2 12893414

[B41] Hafke H. Z. (2008). Nonconscious control of fundamental voice frequency. J. Acoust. Soc. Am. 123, 273–278 10.1121/1.2817357 18177157

[B42] Halpern A. R. Zatorre R. J. (1979). Identification, discrimination, and selective adaptation of simultaneous musical intervals. Percept. Psychophys. 26, 384–395 10.3758/BF03204164 523282

[B43] Heilman K. M. Voeller K. Alexander A. W. (1996). Developmental dyslexia: a motor-articulatory feedback hypothesis. Ann. Neurol. 39, 407–412 10.1002/ana.410390323 8602765

[B44] Heyes C. (2011). Automatic imitation. Psychol. Bull. 137, 463–483 10.1037/a0022288 21280938

[B45] Hickok G. (2009). Eight problems for the mirror neuron theory of action understanding in monkeys and humans. J. Cogn. Neurosci. 21, 1229–1243 10.1162/jocn.2009.21189 19199415 PMC2773693

[B46] Hickok G. Poeppel D. (2004). Dorsal and ventral streams: a framework for understanding aspects of the functional anatomy of language. Cognition 92, 67–99 10.1016/j.cognition.2003.10.011 15037127

[B47] Hickok G. Poeppel D. (2007). The cortical organization of speech processing. Nat. Rev. Neurosci. 8, 393–402 10.1038/nrn2113 17431404

[B48] Hutchins S. Peretz I. (2010). Double dissociation of pitch production and perception, in Presented at the Seventeenth Annual Meeting of the Cognitive Neuroscience Society, (Montreal, QC), 127. Available online at: http://www.cogneurosociety.org/wordpress/wp-content/themes/CNStheme/downloads/CNS2010_Program.pdf 10.1016/j.neuropsychologia.2011.11.015

[B49] Hutchins S. Peretz I. (2012a). A frog in your throat or in your ear. Studying the causes of poor singing. J. Exp. Psychol. Gene. 141, 76–97 10.1037/a0025064 21875245

[B50] Hutchins S. Peretz I. (2012b). Amusics can imitate what they cannot discriminate. Brain Lang, 123, 234–239 10.1016/j.bandl.2012.09.011 23117156

[B51] Hutchins S. Peretz I. (2013). Vocal pitch shift in congenital amusia (pitch deafness). Brain Lang. 125, 106–117 10.1016/j.bandl.2013.01.011 23467261

[B52] Hutchins S. Roquet C. Peretz I. (2012). The vocal generosity effect: how bad can your singing be. Music Percept. 30, 147–159 10.1525/mp.2012.30.2.147

[B53] Hutchins S. Zarate J. M. Zatorre R. J. Peretz I. (2010). An acoustical study of vocal pitch matching in congenital amusia. J. Acoust. Soc. Am. 127, 504–512 10.1121/1.3270391 20058995

[B54] Hyde K. L. Lerch J. P. Zatorre R. J. Griffiths T. D. Evans A. C. Peretz I. (2007). Cortical thickness in congenital amusia: when less is better than more. J. Neurosci. 27, 13028–13032 10.1523/JNEUROSCI.3039-07.2007 18032676 PMC6673307

[B55] Hyde K. L. Peretz I. (2004). Brains that are out of tune but in time. Psycholog. Sci. 15, 356–360 10.1111/j.0956-7976.2004.00683.x 15102148

[B56] Hyde K. L. Zatorre R. J. Peretz I. (2011). Functional MRI evidence of an abnormal neural network for pitch processing in congenital amusia. Cereb. Cortex 21, 292–299 10.1093/cercor/bhq094 20494966

[B57] Kleber B. Birbaumer N. Veit R. Trevorrow T. Lotze M. (2007). Overt and imagined singing of an Italian aria. Neuroimage 36, 889–900 10.1016/j.neuroimage.2007.02.053 17478107

[B58] Kleber B. Veit R. Birbaumer N. Gruzelier J. Lotze M. (2010). The brain of opera singers: experience-dependent changes in functional activation. Cereb. Cortex 20, 1144–1152 10.1093/cercor/bhp177 19692631

[B59] Kohler E. Keysers C. Umiltà M. A. Fogassi L. Gallese V. Rizzolatti G. (2002). Hearing sounds, understanding actions: action representation in mirror neurons. Science 297, 846–848 10.1126/science.1070311 12161656

[B60] Kraus N. Skoe E. Parbery-Clark A. Ashley R. (2009). Experience-induced malleability in neural encoding of pitch, timbre, and timing. Ann. N.Y. Acad. Sci. 1169, 543–557 10.1111/j.1749-6632.2009.04549.x 19673837 PMC2810198

[B61] Krishnan A. (2007). Human frequency following response, in Auditory Evoked Potentials: Basic Principles and Clinical Application, eds Burkard R. F. Don M. Eggermont J. J. (Baltimore, MD: Lippincott Williams and Wilkins), 313–335

[B62] Krishnan A. Swaminathan J. Gandour J. T. (2008). Experience-dependent enhancement of linguistic pitch representation in the brainstem is not specific to a speech context. J. Cogn. Neurosci. 21, 1092–1105 10.1162/jocn.2009.21077 18702588 PMC4373537

[B63] Krumhansl C. L. Iverson P. (1992). Perceptual interactions between musical pitch and timbre. J. Exp. Psychol. Hum. Percept. Perform. 18, 739–751 10.1037/0096-1523.18.3.739 1500873

[B64] Léveque Y. Giovanni A. Schön D. (2012). Pitch-matching in poor singers: human model advantage. J. Voice 26, 293–298 10.1016/j.jvoice.2011.04.001 21816572

[B65] Levitin D. J. Rogers S. E. (2005). Absolute pitch: perception, coding, and controversies. Trends Cogn. Sci. 9, 26–33 10.1016/j.tics.2004.11.007 15639438

[B66] Levy D. A. Granot R. Bentin S. (2001). Processing specificity for human voice stimuli: electrophysiological evidence. Neuroreport 12, 2653–2657 10.1097/00001756-200108280-00013 11522942

[B67] Liberman A. M. Mattingly I. G. (1985). The motor theory of speech perception revised. Cognition 21, 1–36 10.1016/0010-0277(85)90021-6 4075760

[B68] Liu F. Patel A. D. Fourcin A. Stewart L. (2010). Intonation processing in congenital amusia: discrimination, identification and imitation. Brain 133, 1682–1693 10.1093/brain/awq089 20418275

[B69] Loui P. Alsop D. Schlaug G. (2009). Tone deafness: a new disconnection syndrome. J. Neurosci. 29, 10215–10220 10.1523/JNEUROSCI.1701-09.2009 19692596 PMC2747525

[B70] Loui P. Guenther F. H. Mathys C. Schlaug G. (2008). Action–perception mismatch in tone-deafness. Curr. Biol. 18, R331–R332 10.1016/j.cub.2008.02.045 18430629 PMC2791531

[B71] Mandell J. Schulze K. Schlaug G. (2007). Congenital amusia: an auditory-motor feedback disorder. Restor. Neurol. Neurosci. 25, 323–334 17943009

[B72] Marin M. M. Gingras B. Stewart L. (2012). Perception of musical timbre in congenital amusia: categorization, discrimination and short-term memory. Neuropsychologia 50, 367–378 10.1016/j.neuropsychologia.2011.12.006 22201556

[B73] Marques C. Moreno S. Besson M. (2007). Musicians detect pitch violation in a foreign language better than nonmusicians: behavioral and electrophysiological evidence. J. Cogn. Neurosci. 19, 1453–1463 10.1162/jocn.2007.19.9.1453 17714007

[B74] Marslen-Wilson W. (1973). Linguistic structure and speech shadowing at very short latencies. Nature. 244, 522–523 10.1038/244522a0 4621131

[B75] Marslen-Wilson W. (1985). Speech shadowing and speech comprehension. Speech Commun. 4, 55–73 10.1016/0167-6393(85)90036-6

[B76] McDermott J. H. Lehr A. J. Oxenham A. J. (2010). Individual differences reveal the basis of consonance. Curr. Biol. 20, 1035–1041 10.1016/j.cub.2010.04.019 20493704 PMC2885564

[B77] Melara R. D. Marks L. E. (1990a). HARD and SOFT interacting dimensions: differential effects of dual context on classification. Percept. Psychophys. 47, 307–325 10.3758/BF03210870 2345683

[B78] Melara R. D. Marks L. E. (1990b). Interaction among auditory dimensions: timbre, pitch, and loudness. Percept. Psychophys. 48, 169–178 10.3758/BF03207084 2385491

[B79] Melara R. D. Marks L. E. (1990c). Perceptual primacy of dimensions: support for a model of dimensional interaction. J. Exp. Psychol. Hum. Percept. Perform. 16, 398–414 10.1037/0096-1523.16.2.398 2142208

[B80] Mellody M. Wakefield G. H. (2000). The time-frequency characteristics of violin vibrato: modal distribution analysis and synthesis. J. Acoust. Soc. Am. 107, 598 10.1121/1.428326 10641668

[B81] Moore R. E. Estis J. Gordon-Hickey S. Watts C. (2008). Pitch discrimination and pitch matching abilities with vocal and nonvocal stimuli. J. Voice 22, 399–407 10.1016/j.jvoice.2006.10.013 17509827

[B82] Moore R. E. Keaton C. Watts C. (2007). The role of pitch memory in pitch discrimination and pitch matching. J. Voice 21, 560–567 10.1016/j.jvoice.2006.04.004 16730946

[B83] Moreau P. Jolicoeur P. Peretz I. (2009). Automatic brain responses to pitch changes in congenital amusia. Ann. N.Y. Acad. Sci. 1169, 191–194 10.1111/j.1749-6632.2009.04775.x 19673779

[B84] Moreau P. Jolicoeur P. Peretz I. (2013). Pitch discrimination without awareness in congenital amusia: evidence from event-related potentials. Brain Cogn. 81, 337–344 10.1016/j.bandc.2013.01.004 23434917

[B85] Moreno S. Besson M. (2006). Musical training and language-related brain electrical activity in children. Psychophysiology 43, 287–291 10.1111/j.1469-8986.2006.00401.x 16805867

[B86] Moreno S. Bidelman G. M. (2013). Examining neural plasticity and cognitive benefit through the unique lens of musical training. Hear. Res. [Epub ahead of print]. 10.1016/j.heares.2013.09.012 24079993

[B87] Moreno S. Marques C. Santos A. Santos M. Besson M. (2009). Musical training influences linguistic abilities in 8-year-old children: more evidence for brain plasticity. Cereb. Cortex 19, 712–723 10.1093/cercor/bhn120 18832336

[B88] Näätänen R. Paavilainen P. Rinne T. Alho K. (2007). The mismatch negativity (MMN) in basic research of central auditory processing: a review. Clin. Neurophysiol. 118, 2544–2590 10.1016/j.clinph.2007.04.026 17931964

[B89] Nikjeh D. A. Lister J. J. Frisch S. A. (2009). Preattentive cortical-evoked responses to pure tones, harmonic tones, and speech: influence of music training. Ear Hear. 30, 432–446 10.1097/AUD.0b013e3181a61bf2 19494778

[B90] Özdemir E. Norton A. Schlaug G. (2006). Shared and distinct neural correlates of singing and speaking. Neuroimage 33, 628–635 10.1016/j.neuroimage.2006.07.013 16956772

[B91] Patel A. (2011). Why would musical training benefit the neural encoding of speech. The OPERA hypothesis. Front. Psychol. 2:142 10.3389/fpsyg.2011.00142 21747773 PMC3128244

[B92] Peretz I. (2008). Musical disorders from behavior to genes. Curr. Dir. Psycholog. Sci 17, 329–333 10.1111/j.1467-8721.2008.00600.x16970864

[B93] Peretz I. Ayotte J. Zatorre R. J. Mehler J. Ahad P. Penhune V. B. (2002). Congenital amusia: a disorder of fine-grained pitch discrimination. Neuron 33, 185–191 10.1016/S0896-6273(01)00580-3 11804567

[B94] Peretz I. Brattico E. Järvenpää M. Tervaniemi M. (2009). The amusic brain: in tune but unaware. Brain 132, 1277–1286 10.1093/brain/awp055 19336462

[B95] Peretz I. Champod A. S. Hyde K. (2003). Varieties of musical disorders. Ann. N.Y. Acad. Sci. 999, 58–75 10.1196/annals.1284.006 14681118

[B96] Peretz I. Cummings S. Dubé M. P. (2007). The genetics of congenital amusia (tone deafness): a family-aggregation study. Am. J. Hum. Genet. 81, 582–588 10.1086/521337 17701903 PMC1950825

[B97] Perry D. W. Zatorre R. J. Petrides M. Alivisatos B. Meyer E. Evans A. C. (1999). Localization of cerebral activity during simple singing. Neuroreport 10, 3979–3984 10.1097/00001756-199912160-00046 10716244

[B98] Pitt M. A. (1994). Perception of pitch and timbre by musically trained and untrained listeners. J. Exp. Psychol. Hum. Percept. Perform. 20, 976 10.1037/0096-1523.20.5.976 7964532

[B99] Pitt M. A. Crowder R. G. (1992). The role of spectral and dynamic cues in imagery for musical timbre. J. Exp. Psychol. Hum. Percept. Perform. 18, 728 10.1037/0096-1523.18.3.728 1500872

[B100] Pfordresher P. Q. Brown S. (2007). Poor-pitch singing in the absence of “tone deafness”. Music Percept. 25, 95–115 10.1525/mp.2007.25.2.95 21811479

[B101] Pfordresher P. Q. Brown S. (2009). Enhanced production and perception of musical pitch in tone language speakers. Attent. Percept. Psychophys. 71, 1385–1398 10.3758/APP.71.6.1385 19633353

[B102] Pfordresher P. Q. Brown S. Meier K. M. Belyk M. Liotti M. (2010). Imprecise singing is widespread. J. Acoust. Soc. Am. 128, 2182 10.1121/1.3478782 20968388

[B103] Prame E. (1997). Vibrato extent and intonation in professional Western lyric singing. J. Acoust. Soc. Am. 102, 616 10.1121/1.419735

[B104] Rakowski A. (1990). Intonation variants of musical intervals in isolation and in musical contexts. Psychol. Music 18, 60–72 10.1177/0305735690181005

[B105] Rizzolatti G. Arbib M. A. (1998). Language within our grasp. Trends Neurosci. 21, 188–194 10.1016/S0166-2236(98)01260-0 9610880

[B106] Rizzolatti G. Craighero L. (2004). The mirror-neuron system. Annu. Rev. Neurosci. 27, 169–192 10.1146/annurev.neuro.27.070203.144230 15217330

[B107] Rizzolatti G. Fogassi L. Gallese V. (2001). Neurophysiological mechanisms underlying the understanding and imitation of action. Nat. Rev. Neurosci. 2, 661–670 10.1038/35090060 11533734

[B108] Sataloff R. T. (2005). Professional Voice: the Science and Art of Clinical Care. San Diego, CA: Plural Publishing

[B109] Seashore C. E. (1938). Psychology of Music. New York, NY: McGraw-Hill

[B110] Shonle J. I. Horan K. E. (1980). The pitch of vibrato tones. J. Acoust. Soc. Am. 67, 246 10.1121/1.383733 7354192

[B111] Siegel J. A. Siegel W. (1977). Categorical perception of tonal intervals: musicians can't tell sharp from flat. Percept. Psychophys. 21, 399–407 10.3758/BF03199493

[B112] Skoe E. Kraus N. (2010). Auditory brain stem response to complex sounds: a tutorial. Ear Hear. 31, 302–324 10.1097/AUD.0b013e3181cdb272 20084007 PMC2868335

[B113] Slevc L. R. Miyake A. (2006). Individual differences in second-language proficiency does musical ability matter. Psycholog. Sci. 17, 675–681 10.1111/j.1467-9280.2006.01765.x 16913949

[B114] Strait D. L. Chan K. Ashley R. Kraus N. (2012). Specialization among the specialized: auditory brainstem function is tuned in to timbre. Cortex 48, 360–362 10.1016/j.cortex.2011.03.015 21536264

[B115] Sundberg J. (1979). Perception of singing. Speech Transm. Lab. Q. Prog. Status Rep. 20, 1–48

[B116] Sundberg J. (1982). In tune or not. A study of fundamental frequency in music practise. Speech Transm. Lab. Q. Prog. Status Rep. 23, 49–78

[B117] Sundberg J. (1987). The Science of the Singing Voice. Dekalb, IL: Northern Illinois University Press

[B118] Sundberg J. Gramming P. Lovetri J. (1993). Comparisons of pharynx, source, formant, and pressure characteristics in operatic and musical theatre singing. J. Voice 7, 301–310 10.1016/S0892-1997(05)80118-3 8293062

[B119] Sundberg J. Prame E. Iwarsson J. (1996). Replicability and accuracy of pitch patterns in professional singers, in Controlling Complexity and Chaos: 9th Vocal Fold Physiology Symposium, (San Diego, CA: Singular Press), 291–306

[B120] Sundberg J. Thalén M. (2010). What is “Twang”. J. Voice 24, 654–660 10.1016/j.jvoice.2009.03.003 20083379

[B121] Takeuchi A. H. Hulse S. H. (1993). Absolute pitch. Psychol. Bull. 113, 345 10.1037/0033-2909.113.2.345 8451339

[B122] Tillmann B. Schulze K. Foxton J. M. (2009). Congenital amusia: a short-term memory deficit for non-verbal, but not verbal sounds. Brain Cogn. 71, 259–264 10.1016/j.bandc.2009.08.003 19762140

[B123] Titze I. R. (2000). Principles of Voice Production. (Second Printing). Iowa, IA: National Center for Voice and Speech

[B124] Tremblay-Champoux A. Dalla Bella S. Phillips-Silver J. Lebrun M. A. Peretz I. (2010). Singing proficiency in congenital amusia: imitation helps. Cogn. Neuropsychol. 27, 463–476 10.1080/02643294.2011.567258 21864199

[B125] van Besouw R. M. Brereton J. S. Howard D. M. (2008). Range of tuning for tones with and without vibrato. Music Percept. 26, 145–155 10.1525/mp.2008.26.2.145

[B126] Vurma A. (2010). Mistuning in two-part singing. Logoped. Phoniatr. Vocol. 35, 24–33 10.3109/14015430903581591 20350073

[B127] Vurma A. Ross J. (2006). Production and perception of musical intervals. Music Percept. 23, 331–344 10.1525/mp.2006.23.4.331

[B128] Warrier C. M. Zatorre R. J. (2002). Influence of tonal context and timbral variation on perception of pitch. Percept. Psychophys. 64, 198–207 10.3758/BF03195786 12013375

[B130] Watts C. R. Hall M. D. (2008). Timbral influences on vocal pitch-matching accuracy. Logoped. Phoniatr. Vocol. 33, 74–82 10.1080/14015430802028434 18569646

[B129] Watts C. Barnes-Burroughs K. Adrianopoulos M. Carr M. (2003a). Potential factors related to untrained singing talent: a survey of singing pedagogues. J. Voice 17, 298–307 10.1067/S0892-1997(03)00068-7 14513953

[B132] Watts C. Murphy J. Barnes-Burroughs K. (2003b). Pitch matching accuracy of trained singers, untrained subjects with talented singing voices, and untrained subjects with nontalented singing voices in conditions of varying feedback. J. Voice 17, 185–194 10.1016/S0892-1997(03)00023-7 12825651

[B131] Watts C. Moore R. McCaghren K. (2005). The relationship between vocal pitch-matching skills and pitch discrimination skills in untrained accurate and inaccurate singers. J. Voice 19, 534–543 10.1016/j.jvoice.2004.09.001 16301100

[B133] Williamson V. J. McDonald C. Deutsch D. Griffiths T. D. Stewart L. (2010). Faster decline of pitch memory over time in congenital amusia. Adv. Cogn. Psychol. 6, 15–22 10.2478/v10053-008-0073-5 20689638 PMC2916665

[B134] Yarbrough C. Green G. A. Benson W. Bowers J. (1991). Inaccurate singers: an exploratory study of variables affecting pitch-matching. Bull. Counc. Res. Music Educ. 107, 23–34

[B135] Yoo L. Sullivan D. S. Jr. Moore S. Fujinaga I. (1998). The effect of vibrato on response time in determining the pitch relationship of violin tones, in Proceedings of the 5th International Conference on Music Perception and Cognition. (Seoul), 209–211

[B136] Zarate J. M. Delhommeau K. Wood S. Zatorre R. J. (2010a). Vocal accuracy and neural plasticity following micromelody-discrimination training. PLoS ONE 5:e11181 10.1371/journal.pone.0011181 20567521 PMC2887372

[B137] Zarate J. M. Wood S. Zatorre R. J. (2010b). Neural networks involved in voluntary and involuntary vocal pitch regulation in experienced singers. Neuropsychologia 48, 607–618 10.1016/j.neuropsychologia.2009.10.025 19896958

[B138] Zarate J. M. Zatorre R. J. (2008). Experience-dependent neural substrates involved in vocal pitch regulation during singing. Neuroimage 40, 1871–1887 10.1016/j.neuroimage.2008.01.026 18343163

[B139] Zwicker E. Fastl H. (1999). Psychoacoustics: Facts and models, Vol. 2. Berlin: Springer 10.1007/978-3-662-09562-1

